# The Effect of miRNA Gene Regulation on HIV Disease

**DOI:** 10.3389/fgene.2022.862642

**Published:** 2022-05-04

**Authors:** Romona Chinniah, Theolan Adimulam, Louansha Nandlal, Thilona Arumugam, Veron Ramsuran

**Affiliations:** ^1^ Centre for the AIDS Programme of Research in South Africa (CAPRISA), University of KwaZulu-Natal, Durban, South Africa; ^2^ School of Laboratory Medicine and Medical Sciences, University of KwaZulu-Natal, Durban, South Africa

**Keywords:** microRNA, HIV, host-genetics, epigenetics, miRNA

## Abstract

Over many years, research on HIV/AIDS has advanced with the introduction of HAART. Despite these advancements, significant gaps remain with respect to aspects in HIV life cycle, with specific attention to virus-host interactions. Investigating virus-host interactions may lead to the implementation of novel therapeutic strategies against HIV/AIDS. Notably, host gene silencing can be facilitated by cellular small non-coding RNAs such as microRNAs paving the way for epigenetic anti-viral therapies. Numerous studies have elucidated the importance of microRNAs in HIV pathogenesis. Some microRNAs can either promote viral infection, while others can be detrimental to viral replication. This is accomplished by targeting the HIV-proviral genome or by regulating host genes required for viral replication and immune responses. In this review, we report on 1) the direct association of microRNAs with HIV infection; 2) the indirect association of known human genetic factors with HIV infection; 3) the regulation of human genes by microRNAs in other diseases that can be explored experimentally to determine their effect on HIV-1 infection; and 4) therapeutic interactions of microRNA against HIV infection.

## 1 Introduction

The Human Immunodeficiency Virus (HIV) is a member of the lentivirus family of retroviruses that infects humans and increases susceptibility to Acquired Immunodeficiency Syndrome (AIDS). At the end of 2020, more than 38 million people were living with HIV globally (Global, 2020). While an effective vaccine remains elusive, extensive research on the inhibition of various stages of the HIV life cycle has paved the way for the development of many antiretroviral drugs (Cohen et al., 2016). Despite the progress with lifesaving, highly active antiretroviral therapy (HAART), treatment may lead to the development of drug toxicities and resistance (Pomerantz and Horn, 2003). HAART has also been implicated in the onset of adverse metabolic effects such as dyslipidaemia, elevated blood pressure, and insulin resistance (Palios et al., 2011). These compounding factors emphasise the necessity for new less toxic, more effective and additional, complementary therapeutic approaches.

Advancements in discovering and determining the function of host factors in viral biogenesis and transmission highlight the possibility of developing new therapeutic tools for preventative measures and treatment of HIV/AIDS (Hoxie and June, 2012). As such, modulating gene expression post-transcriptionally using small non-coding RNAs (sncRNAs) mediates cellular gene silencing through RNA interference (RNAi). This mode of regulation has become increasingly utilized in the development and delivery of the therapeutic anti-viral strategy (Balasubramaniam et al., 2018). Eukaryotic cells possess endogenous RNAi mechanisms, of which microRNAs (miRNAs) are the most significant family of sncRNAs (Ghildiyal and Zamore, 2009). MiRNAs are a class of small non-coding RNA molecules (21–25 nucleotides in length) that are instrumental in regulating gene expression of multiple cellular processes, including differentiation, development, apoptosis, and stress response (Felekkis et al., 2010). These molecules exert their regulatory mechanisms by mRNA degradation or translational repression (prevention of translation of target mRNAs) (Cai et al., 2009; Fabian et al., 2010; Inui et al., 2010; Subramanian and Steer, 2010). The biogenesis of miRNAs is detailed profoundly in several manuscripts, which describe the two principal pathways (canonical and non-canonical) (O'Brien et al., 2018; Ha and Kim, 2014; Macfarlane and R. Murphy, 2010; Zhao et al., 2019).

Briefly, the canonical pathway begins in the nucleus where a primary RNA (pri-miRNA), usually ∼80 nucleotides long, is transcribed from its specific gene by RNA polymerase II. The pri-miRNA is then cleaved to form a precursor miRNA (pre-miRNA), generally ∼60 nucleotides long, by the Microprocessor complex (Zhao et al., 2019). The Multiprocessor complex consists of two multiprotein units. The first is a large multiprotein unit. The second is a small multiprotein which constitutes of Drosha (RNase III enzyme) and the RNA binding protein DiGeorge Syndrome Critical Region 8 (DGCR8) (Gregory et al., 2004). Once the pre-miRNA is generated, it is transported to the cytoplasm by exportin-5 and Ran-GTP, where it undergoes cleavage by Dicer (O'Brien et al., 2018). The Dicer enzyme removes the terminal loop, thus resulting in a double-stranded product that consists of the mature miRNA guide strand and a passenger strand. The mature miRNA product will be transferred onto Argonaute (AGO) protein (Macfarlane and R. Murphy, 2010). The remaining passenger strands are usually directed toward degradation. However, the guide strand is further integrated into the RNA-induced silencing complex (RISC) (O'Brien et al., 2018; Macfarlane and R. Murphy, 2010). Finally, the RISC-miRNA complex principally binds to the 3′UTR of the target mRNA. The complementarity of this binding predicts the fate of the mRNA, such that, in the event of perfect complementarity, the target mRNA is degraded. However, when this binding is incomplete, the mRNA is translationally repressed (Cai et al., 2009).

Several non-canonical pathways have been described (Annese et al., 2020). In summary, non-canonical pathways are classified into Drosha/DGCR8-independent and Dicer-independent pathways. The class of Drosha/DGCR8-independent miRNAs which originate from spliced introns are commonly known as mirtrons. These miRNAs are instantly transported to the cytoplasm via Dicer processing (Treiber et al., 2019). On the contrary, Dicer-independent miRNAs are uncommon. Drosha processes Dicer-independent miRNAs from endogenous short hairpin RNA (shRNA) transcripts, directly recognised by Ago proteins, thus making them Dicer-independent (Dai et al., 2019).

Multiple studies have linked aberrant miRNA profiles to diseases such as cancer (Croce and Calin, 2005; Calin and Croce, 2006), neurodegenerative disease (Kim et al., 2007; Wang et al., 2008), autoimmune disease (Dai et al., 2007; Stanczyk et al., 2008; Zhao et al., 2010), inflammatory diseases (Sonkoly et al., 2007), muscular disorders (Eisenberg et al., 2007), cardiovascular disorders (Carè et al., 2007; Ikeda et al., 2007), in addition to developmental abnormalities and psychiatric disorders (Lewis et al., 2003). Moreover, the five biggest infectious killers globally, including HIV/AIDS, are responsible for approximately 80% of the total contagious disease burden. About 12 million people per year succumb to these diseases, primarily in developing countries (Organization, 2020). Comparable to non-infectious conditions, miRNAs affect host and virus interactions in various ways. They are characterised as direct alteration of viral replication by influencing viral susceptibility or as indirect alteration of host genes that influence viral replication (Scaria et al., 2007; Kumar and Jeang, 2008).

MiRNAs have previously been implicated in HIV infection (Sun et al., 2016; Balasubramaniam et al., 2018; Su et al., 2018). As a field in its infancy, there is a substantial benefit in determining the impact of miRNAs on HIV infection.

This review discusses the direct alterations of miRNAs in HIV infection and the indirect alterations of known human genetic factors in HIV infection. Thereafter, we describe miRNA associations of known human genetic factors with other diseases that can be exploited to determine their specific effect on HIV infection, and the potential use of miRNAs as therapeutic interactions against HIV infection.

## 2 Effect of miRNAs on HIV Infection

**FIGURE 1:**
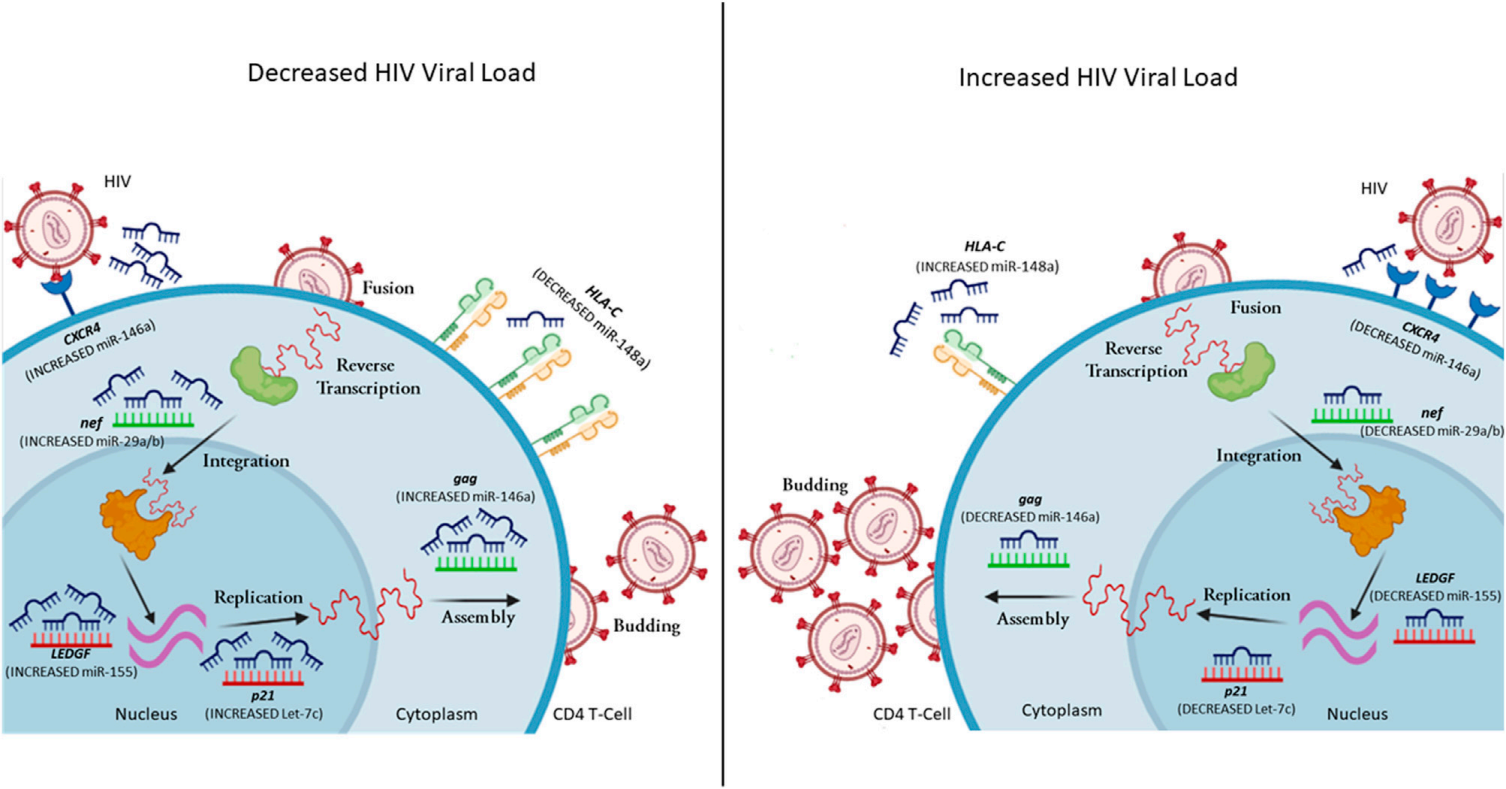
A representation of selected miRNAs that control gene expression levels, leading to variability in HIV viral load. MiRNA can regulate both host (red mRNA) and viral (green mRNA) mRNA. In the case of decreased viral load, the CD4+ T cell has increased expression of miR-146a (reduces *CXCR4* and *gag* expression), miR-29a/b (reduces *nef* expression), miR-155 (reduces *LEDGF* expression), Let-7c (reduces *p21* expression), while decreased expression of miR-148a upregulates HLA-C expression. In the case of increased viral load, the CD4+ T cell has decreased expression of miR-146a (increases *CXCR4* and *gag* expression), miR-29a/b (increases *nef* expression), miR-155 (increases *LEDGF* expression), Let-7c (increases *p21* expression), while increased expression of miR-148a down-regulates *HLA-C* expression (complied using BioRender).

MiRNAs can aid or obstruct HIV infection at various stages of the viral life cycle, affecting viral replication, host immune response, and ultimately disease management (Figure 1). HIV exploits and uses cellular miRNAs to modulate its replication by directly targeting its RNA or host mRNAs that would negatively impact HIV replication. In addition, miRNAs are linked with a possible susceptibility to HIV infection in monocytes and macrophages (Wang et al., 2009; Qiuling et al., 2018). Furthermore, the viral genome may produce viral encoded miRNAs that modulate viral RNAs as well as cellular mRNAs (Cullen, 2006; Skalsky and Cullen, 2010). This suggests that HIV could potentially regulate its replication cycle and possibly program its own latency (Omoto et al., 2004; Bennasser et al., 2006; Ouellet et al., 2013; Zhang et al., 2014). Several cellular miRNAs have demonstrated the ability to modulate HIV infection, either directly or indirectly (Table 1).

**TABLE 1:** Studies showing microRNAs affecting host cell genes in the context of HIV infection.

microRNA	Target	Action	Experimental approach/observation	References number
miR-148a	*HLA-C*	Impaired control of HIV viral load	*In vitro* studies Genetic association with HIV P = 2 × 10^−14,^ R = 0.33^,^N = 2.527 (European cohort)	Kulkarni et al. (2011)
miR-146a	*CXCR4*	Prevents HIV entry	*In vitro*	Quaranta et al. (2015)
miR-132	*MeCP2*	Enhances HIV infection	*In vitro*	Chiang et al. (2013)
miR-182	*NAMPT*	Enhance HIV tat-mediated trans-activation	*In vitro*	Chen et al. (2013)
miR-34a miR-217	*SIRT1*	Enhances HIV tat mediated trans-activation	*In vitro In vitro*	Zhang et al. (2012a), Zhang et al. (2012b)
miR-34a	*PNUTS*	Promotes HIV -1 transcription	*In vitro*	Kapoor et al. (2015)
miR- 155	*TRIM32*	Promotes reactivation of latent HIV via NF-kB signalling	*In vitro*	Ruelas et al. (2015)
miR-17-5p miR-20a	*PCAF*	Reduction of HIV infection	*In vitro*	Triboulet et al. (2007)
miR-198 miR-27b miR-29b miR-150 miR-223	*Cyclin T1*	Impaired replication in monocytes Impaired HIV replication in resting CD4+ T cells	*In vitro In vitro*	Sung and Rice. (2009), Chiang et al. (2012)
miR-15a miR-15b miR-16 miR-20a miR-93 miR-106b	*Pur-Alpha*	Impaired HIV replication in monocytes	*In vitro*	Shen et al. (2012)
miR-155	*ADAM 10*	Reduction of HIV late RT products and viral DNA integration in MDM	*In vitro*	Swaminathan et al. (2012c)
miR-155	*NUP153*	Reduction of HIV late RT products and viral DNA integration in MDM	*In vitro*	Swaminathan et al. (2012c)
miR-155	*LEDGF/p75*	Reduction of HIV late RT products and viral DNA integration in MDM	*In vitro*	Swaminathan et al. (2012c)
miR-155 miR-181	*SAMHD1*	Overexpression of miR-155/181a enhanced HIV replication in astrocytes	*In vitro*	Pilakka-Kanthikeel et al. (2015)
miR1236	*VprBP*	Impaired HIV replication in monocytes	*In vitro*	Ma et al. (2014)
let-7c	*p21*	Increased HIV replication	*In vitro*	Farberov et al. (2015)
miR-124a miR34a-5p	*TASK1*	Increased HIV replication	*In vitro*	Farberov et al. (2015)
miR-146a	*CCL5*	Enhance HIV infection	*In vitro*	Qiuling et al. (2018)
miR-21	*IP-10*	miR-21 expression downregulates IP-10 controlling the loss of CD4+ T cells which is closely related to disease progression	Genetic association in HIV disease *p* < 0.0001, R = 0.706, N = 32 (Chinese cohort)	Wu et al. (2017)
miR-155	*PU.1 (DC-SIGN)*	Reduces HIV entry into T lymphocytes	*In vitro*	Martinez-Nunez et al. (2009)
miR-9	*BLIMP-1*	Reduced HIV infection	*Ex vivo and in vitro*	Seddiki et al. (2013)
let-7	*IL-10*	Reduced HIV infection	*Ex vivo and in vitro*	Swaminathan et al. (2012c)
miR-221 miR-222	*CD4*	Inhibition of HIV entry in macrophages	*In vitro*	Lodge et al. (2017)
miR-34c-5p	Several genes are involved in TCR signaling and activation of naïve CD4^+^ T cells	Increased HIV replication	*In vitro*	Amaral et al. (2017)
miR-29a miR-29b miR-29c	*IL-32*	Proviral load and disease progression	Genetic association in HIV disease *p* = 0.079, R = 0.232, N = 58 *p* = 0.102, R = 0.445, N = 58 *p* = 0.103, R = 0.216, N = 58	Monteleone et al. (2015)

Notes: P represents the p value for the specific result. R represents the value of the statistical Pearson R. N is representative of the number of samples. The italic values under the “Target” column is indicative of gene names. While the italic values under the “Experimental approach/observation” is the Latin caption used to define how the experiment was performed.

### 2.1 Regulation of HIV Replication Through Viral Genome

Host derived miRNAs can bind to HIV RNA, directly regulating pathogenesis (Trobaugh and Klimstra, 2017). For instance, recent data has shown that miR-139-5p plays a role in activating latent HIV infected cells, by regulating *FOX01*, as well as FOS and JUN transcription factors (Okoye et al., 2021). The expression of miR-28, miR-125b, miR-150, miR-223, and miR-382 were significantly lower in activated CD4+ T cells in comparison to its resting counterpart. The same group of miRNAs may play a role in establishing viral latency by interacting with a conserved 1.2 kb fragment found in the 3′UTR of all HIV transcripts. These miRNAs can inhibit the translation of all viral proteins with the exception of *nef* (Huang et al., 2007). Moreover, the study showed that infected cells with established latency could be reactivated by treatment with miRNA inhibitors, suggesting that cellular miRNAs may provide a mechanistic effect towards HIV latency (Huang et al., 2007). Besides their role in promoting HIV latency, these five miRNAs play a crucial role in preventing HIV infection of monocytes and monocyte-derived macrophages (MDM). MiR-28, miR-125b, miR-150, miR-223, and miR-382 were observed at significantly higher levels in monocytes compared to MDM. These miRNAs were found to impede HIV reverse transcriptase activity in both cell types. However, the activity of HIV reverse transcriptase was dependant on the level of these miRNAs. This may explain why monocyte differentiation into macrophages is required for effective HIV infection (Wang et al., 2009).

*Nef* expression can also be influenced by cellular miRNAs (Ahluwalia et al., 2008; Sun et al., 2012). Ahluwalia *et al.* found that miR-29a and miR-29b may target HIV *nef* expression, which resulted in repression of *nef* translation and subsequent decrease in viral load (Figure 1) (Ahluwalia et al., 2008).

Moreover, in a series of refined experiments, Sun *et al.* demonstrated a new regulatory circuit during HIV infection (Sun et al., 2012). The downregulation of the miR-29 family could be associated with *nef* up-regulation and apoptosis of CD4+ cells (Sun et al., 2012). In addition, previous studies showed that miR-29 inhibited HIV replication by approximately 60%, while miR-133b, miR-138, miR-326, miR-149, and miR-92a reduced HIV viral replication by 40% (Houzet et al., 2012). In silico screening showed that these miRNAs may possibly target the *5′LTR* (miR-326), *env* (miR-133b, miR-138), *gag* (miR-149), and *pol* (miR-92a) leading to the repression of viral replication.

Recent work by Chen *et al.* showed another form of miRNA regulation of HIV viruses through the interaction of miR-146a with the viral protein *gag* (Figure 1) (Chen et al., 2014). This interaction resulted in a viral-RNA-mediated gag assembly blockage, thereby interfering with viral budding and infectivity (Chen et al., 2014). These findings illustrate that miRNAs can alter viral gene expression via direct targeting of HIV mRNAs, with variable mechanisms of action dictated by the cell types.

### 2.2 Host Factors That Regulate HIV Replication

MiRNAs regulate HIV infection through indirect modulation of host factor expression. One viral-dependent factor in cells is Cyclin T1, characterised as an essential part of the PTEFb complex, responsible for facilitating viral transcription (Hoque et al., 2011). The direct modulation is facilitated through the interaction with *tat,* which recruits the complex to HIV TAR, thereby impacting viral latency (Hoque et al., 2011). Recent work by Sung *et al.* described that miR-198targets and down-regulates Cyclin T1 mRNA and protein expression, which subsequently impairs the *tat*-mediated transcriptional activation of HIV in infected monocytes and macrophages (Sung and Rice, 2009). Over-expression of miR-198 inhibited HIV replication in macrophages, suggesting that cell type-specific mechanisms may be an effect executed by miRNAs (Sung and Rice, 2009). Additional studies identified that Cyclin T1 inhibition is exerted by cellular miRNAs (miR-27b, miR-29b, miR-150, and miR-223) in resting CD4+ T cells (Chiang et al., 2012). However, CD4+ T cell activation followed the downregulation of the miRNAs. This result was correlated with enhanced HIV susceptibility and productive replication (Chiang et al., 2012).

The viral protein *tat* is an essential transcriptional activator that interacts with several cellular proteins. For efficient HIV transcriptional activation, *tat* must be acetylated by p300-CREB binding protein associated factor (PCAF) (D'Orso and Frankel, 2009). Remarkably, miR-17/92 family of host miRNAs impedes HIV infection by downregulating PCAF (Triboulet et al., 2007). Triboulet *et al.* also showed that miR-17 as well as miR-20a inhibited PCAF expression at the mRNA and protein levels. In addition, HIV can actively repress miR-17-5p and miR-20a to enhance viral translation through p300/PCAF-dependant *tat* activation (Triboulet et al., 2007).

Another well characterised cellular factor that interacts with HIV *tat* to up-regulate viral transcription is the purine-rich element binding protein α (Pur-α) (Wortman et al., 2000). A collection of six cellular miRNAs (miR-15a, miR-15b, miR-16, miR-20a, miR-93, and miR-106b) enriched in monocytes were linked with the repression of Pur-α (Shen et al., 2012). Consequently, inhibition of these miRNAs in monocytes increased the expression of Pur-α, resulting in an increase in HIV infection (Shen et al., 2012).

MiR-155 has demonstrated significant effects on HIV infection through a Toll-Like receptor (TLR)-dependant mechanism (Swaminathan et al., 2012a). Swaminathan *et al.* showed that miR-155 is significantly up-regulated in MDMs, stimulated by TLR3 and TLR4 (Swaminathan et al., 2012a). Furthermore, up-regulation of miR-155 through TLR stimulation leads to decreased mRNA and protein expression of ADAM10, TNPO3, NUP153, and LEDGF/p75, in MDMs (Swaminathan et al., 2012a). Gene silencing of *LEDGF* had the most significant effect on HIV infection (Figure 1) (Swaminathan et al., 2012a). However, co-silencing of both *LEDGF* and *ADAM10* had a more substantial impact, impairing the transport of viral pre-integration complexes (Swaminathan et al., 2012a).

The inhibition of *TRIM32* by miR-155 results in post-integration latency of HIV (Ruelas et al., 2015). TRIM32 directs NF-κB to the nucleus via a *tat*-independent mechanism, as described by Ruelas et al. (2015). The study characterises a novel mechanism by which TRIM32 activates NF-κB. Collectively, the inhibitory effect of miR-155 on *TRIM32* highlights a new tool for HIV remaining in infected reservoirs (Ruelas et al., 2015). Despite this significant study, recent studies have identified miR-155 as a potent biomarker of activated T cells and immune dysfunction in HIV-infected individuals (Jin et al., 2017a; Jin et al., 2017b; Zhang et al., 2021a).

MiRNAs can also restrict viral entry by targeting the receptors and co-receptors exploited for HIV entry. Orecchini *et al.* report a *tat*-dependant mechanism that controls CD4 receptor by up-regulating miR-222 (Orecchini et al., 2014). In addition, Lodge *et al.* demonstrated that miR-221 and miR-222 are up-regulated in MDMs, targeting the 3′ UTR of CD4 (Orecchini et al., 2014). The mRNA and subsequent protein expression are reduced, ultimately impairing HIV entry into MDM (Lodge et al., 2017). Labbaye *et al.* showed that promyelocytic leukaemia zinc finger (PLZF) could regulate miR-146a, subsequently controlling the expression of *CXCR4 in vitro* (Labbaye et al., 2008). Activation of resting CD4+ T cells by phytohemagglutinin results in the downregulation of miR-146a (Quaranta et al., 2015). Downregulation of miR-146a results in the overexpression of CXCR4 co-receptor promoting viral entry in CD4+ T cells (Quaranta et al., 2015).

Vpr HIV-binding protein (vprBP) is a cellular cofactor that forms part of a ubiquitin protein ligase complex. VprBP promotes HIV infection (Ma et al., 2014). Ma *et al.* demonstrated that miR-1236 inhibitors increased translation of vprBP in monocytes, thus facilitating HIV infection. Contrary to monocytes, miR-1236 mimics in monocyte-derived dendritic cells had supressed vprBP, which was complemented by decreased infection (Ma et al., 2014).

High surface expression of human leukocyte antigen C (HLA-C) greatly corresponded with slower disease progression via superior control of HIV viremia. Several genetic variants have been shown to disrupt miR-148a regulation of HLA-C (Kulkarni et al., 2011; Blais et al., 2012; Kulkarni et al., 2013). Disruption of miRNA binding site allows high expressing HLA-C alleles to escape miR-148a regulation (Kulkarni et al., 2011). HLA-C alleles that do not have a disrupted miR-148a binding site are tightly regulated by miR-148a and are expressed at low levels. The polymorphisms affecting HLA-C expression through disrupted miR-148a binding are rs9264942, rs67384697, and rs735316, with the variants of rs9264942 and rs67384697 being in linkage disequilibrium (Kulkarni et al., 2011; Blais et al., 2012; Kulkarni et al., 2013). All three variants are associated with control and progression of HIV infection by miR-148a-mediated post-transcriptional regulation of HLA-C.

IL-10 is a multifunctional anti-inflammatory cytokine produced by various immune cells. With regards to miRNA regulation of IL-10, the let-7 family can directly target *IL10*. *In vitro* infection with HIV elevated *IL10* levels through the reduction of let-7. In addition, CD4+ T cells of chronically infected HIV-positive individuals had significantly lower let-7 levels than uninfected individuals and long-term non-progressors. (Swaminathan et al., 2012b). A single miRNA is able to regulate multiple target genes. In addition to *IL10*, let-7c is involved in the regulation of *p21*. let-7c overexpression in Jurkat cells resulted in a 1.38-fold change in p21 expression (Figure 1) (Farberov et al., 2015).

B lymphocyte-induced maturation protein-1 (Blimp-1) is a transcriptional repressor of IL-2 (an important cytokine required for T cell growth and survival). In HIV-infected individuals, BLIMP-1 may contribute to T cell dysregulation through alterations in IL-2 levels. MiR-9 inhibited *BLIMP1* expression in CD4+ T cells. Chronically infected HIV-positive patients had lower miR-9 and higher *BLIMP1* expression in comparison to uninfected healthy individuals and long-term non-progressors (Seddiki et al., 2013).

### 2.3 Predicted miRNA Targets for HIV

It is estimated that 1,254 human genes are involved in viral replication. Genome-wide RNA interference has enabled researchers to identify multiple host factors that are involved in HIV life cycle. This large array of host gene targets may be essential in the development of new therapeutic strategies against HIV. By identifying and understanding the mechanisms behind the associations of specific miRNAs and their targets, we can exploit these factors for HIV viral control. Several HIV-associated genes are shown to be under the regulation of miRNAs in other diseases.

Blocking the access of HIV into host cells is the first step in preventing the HIV proviral genome from integrating into the host’s genome. The human chemokine receptor 5 (CCR5) plays an important role in the internalization of HIV into the host cell (Lederman et al., 2006). Individuals with the 32 base pair deletion in their *CCR5* gene are known to be resistant to HIV as they have lower levels of CCR5 on the surface of their CD4+ T cells. Thus, the regulation of CCR5 expression may be essential in inhibiting HIV replication. Che *et al.* found that miR-107 binds to the 3′UTR of *CCR5* (Che et al., 2016). CCR5 proteins and gene expression were found to be significantly lower in the presence of miR-107 (Che et al., 2016). Since CCR5 is important in the HIV context, miR-107 may be of potential therapeutic value in preventing HIV infection.

Intercellular adhesion molecule 1 (ICAM-1) also plays a significant role in HIV entry. The binding of ICAM-1 with LFA-1 on the cell surface facilitates viral infectivity. ICAM-1 increases viral infectivity by directly inserting into mature HIV virions (Fortin et al., 1997; Bounou et al., 2002). Lui et al. demonstrated that *ICAM1* is negatively regulated by miR-296-3p in the malignant highly metastatic M12 cell line (Liu et al., 2013). Furthermore, in prostate cancer cells there is a negative correlation between miR-296-3p and *ICAM1* (Liu et al., 2013). In the context of HIV, the downregulation of *ICAM1* by miR-296-3p would reduce the rate of infectivity (Liu et al., 2013).

The tripartite motif (TRIM) proteins are a family of E3 ubiquitin ligases with diverse anti-viral functions (van Gent et al., 2018). TRIM22, TRIM11, and KAP1 (TRIM28) were previously shown to have anti-HIV activity (Barr et al., 2008; Allouch et al., 2009; Yuan et al., 2016). TRIM22 inhibits the processing of viral particles and viral budding through the ubiquitylation in HIV. TRIM22 also has anti-Hepatitis C virus (HCV) activity. Tian *et al.* confirmed that *TRIM22* was regulated by miR-215 (Tian and He, 2018). In Con1b cells, the overexpression of miR-215 facilitated HCV replication by downregulating *TRIM22.* Knockdown of miR-215 suppressed HCV replication through the increased expression of *TRIM22* in Huh7.5.1 cells (Tian and He, 2018). In colon cancer, *TRIM11* is negatively regulated by miR-24-3p, promoting cellular proliferation and inhibiting apoptosis (Yin et al., 2016). Likewise, Qi *et al.* demonstrated that miR-491 levels inversely corresponded with *TRIM28* expression in glioblastoma multiforme (GBM) (Qi et al., 2016). Their data showed that miR-491 was reduced in GBM and indicated that the low levels of miR-491 are associated with poor prognosis (Qi et al., 2016). miR-491 inhibited TRIM28 translation in GBM cells (Qi et al., 2016).

Studies have also demonstrated a link between *RAD51* expression and HIV disease (Chipitsyna et al., 2004; Cosnefroy et al., 2012; Kaminski et al., 2014; Thierry et al., 2015). Elevated expression of *RAD51* promotes HIV-1 transcription (Kaminski et al., 2014). Evidence demonstrates that *RAD51* may have stimulatory or inhibitory effects on specific steps of retroviral replication cycles (Thierry et al., 2015). These effects depend on RAD51 being able to recruit both transcription machinery and proteins implicated in chromatin remodelling and formulation of RAD51 stimulatory compound (Thierry et al., 2015). Findings from Gasparini *et al.* indicate that DNA repair is indirectly regulated by miR-155 through its interaction with RAD51 in breast cancer (Gasparini et al., 2014).

The regulation of several other HIV-associated host factors such as TRAF6, CCL4, CCL3, IRF7, RSAD2, ISG15, TLR3, SETDB1, and Rab27a by miRNAs could potentially play a role in HIV infection. Table 2 provides a list of HIV-associated host genes which should be investigated in future miRNA studies. The host’s genes and associated miRNAs described in Table 2 may provide novel therapeutic targets against HIV.

**TABLE 2:** Genes associated with HIV infection shown to be regulated by miRNAs in other diseases.

HIV infection			Other disease		
Gene	Effect	References number	microRNA	Disease or infection	References number
Viral receptors					
*CCR5*	responsible for HIV infection and entry	Blanpain et al. (2002); Lederman et al. (2006)	miR-107	Cancer	Che et al. (2016)
*ICAM-1*	assists with HIV entry increasing virus infectivity	Fortin et al., (1997); Bounou et al. (2002)	miR-296-3p	Prostate cancer	Liu et al. (2013)
Innate immune regulators					
*TRIM22*	blocks HIV replication in cell by preventing the assembly of the virus	Barr et al. (2008)	miR-215	HCV	Tian and He, (2018)
*TRIM28 (KAP1)*	inhibits HIV-1 through by targeting the integration step	Allouch et al. (2009)	miR-149	Cancer	Qi et al. (2016)
*TRIM11*	restricts HIV-1 reverse transcription by accelerating viral un-coating	Yuan et al. (2016)	miR-24-3p	Colon cancer	Yin et al. (2016)
*TRAF6*	induced as part of the normal innate immune response against HIV virus	Sirois et al. (2011)	miR-146a miR-144	Dengue virus influenza virus, EMCV, and VSV	Wu et al. (2013) Rosenberger et al. (2017)
T cell exhaustion markers					
*CCL4*	CCR5 ligand involved in blocking HIV entry	Carrol et al. (1999)	miR-125b	Aging	Cheng et al. (2015)
*CCL3*	CCR5 ligand involved in blocking HIV entry	Modi et al., (2006); Levine et al., (2009)	miR-223	Tuberculosis	Dorhoi et al. (2013)
*IRF7*	contributes to enhanced HIV-1 replication	Sirois et al. (2011)	miR-541	Vascular smooth muscle cells	Yang et al. (2016)
*RSAD2* (viperin)	Inhibits viral production	Raposo et al. (2013)	miR-200a miR-200b miR-429	Cell differentiation studies	Li et al. (2016)
*ISG15*	Suppresses HIV replication at various parts of the HIV life cycle	Pincetic et al. (2010); Doyle et al., (2015)	miR-138 miR-370	Oral cancer	Zhang et al. (2017)
Toll-like receptors					
*TLR3*	Innate immune response. Reduces HIV infection	Akira et al., (2006); Swaminathan et al. (2012c)	miR-26a	Arthritis	Jiang et al. (2014)
Other					
*RAD51*	stimulatory or inhibitory effects on specific steps on retroviral replication cycles	(Kaminski et al., 2014; Thierry et al., 2015)	miR-155	Cancer	Gasparini et al. (2014)
*SETDB1*	Inhibits HIV-1 replication at a step prior to integration	(Liu et al., 2011; Tyagi and Kashanchi, 2012)	miR-381-3p	Breast cancer	Wu et al. (2018)
*Rab27a*	Favours HIV assembly	Gerber et al. (2015)	miR-134-3p	Ovarian cancer	Chang et al. (2017)

Notes: HCV, abbreviates Hepatitis C. EMCV, abbreviates encephalomyocarditis virus; VSV, abbreviates vesicular stomatitis virus.

### 2.4 Therapeutic miRNA Targets for HIV

Extensive research has paved the way for developing multiple antiretroviral drugs targeting specific phases of the viral life cycle, leading to a combination of antiretroviral therapy (cART). Currently, this treatment results in controlled viral replication in many treated individuals (Cohen et al., 2016). Despite the progress with lifesaving HAART, infection with HIV remains pathogenic and incurable. In addition, these drugs lead to the development of toxicities and adverse side effects which may only be combated by changing the drug regimen. Furthermore, the increasing emergence of HIV drug resistance poses a threat to the success of the current regimens (Bertagnolio et al., 2012; Le Douce et al., 2012; Stadeli and Richman, 2013). These compounding factors highlight the importance of identifying novel and complementary treatment regimens.

RNA-based therapeutics appear ready to deliver on their promise. Significant success has been observed in several clinical trials using potential miRNA drugs in multiple infectious and non-infectious diseases, including cancer (Hatley et al., 2010; Li et al., 2010; Steele et al., 2011; Wong et al., 2012; Yamanaka et al., 2012), hepatitis C (Jopling et al., 2005; Sarasin-Filipowicz et al., 2009; Lanford et al., 2010), heart abnormalities (Thum et al., 2008; Liu et al., 2010), kidney disease, pathologic fibrosis, and even keloid formation. Interestingly, studies have also shown that dysregulated miRNA profiles play a role in HIV replication (Barr et al., 2008; Pincetic et al., 2010; Liu et al., 2011; Sirois et al., 2011; Tyagi and Kashanchi, 2012; Raposo et al., 2013; Doyle et al., 2015). The vaccine, iHIVARNA is a combination of mRNA sequences that serve as an HIV immunogen. In the first round of clinical trials, iHIVARNA is tolerated in HIV-infected patients on chronic cART (De Jong et al., 2019). Despite this progress, the application of miRNAs as diagnostic and interventional medicine remains an underexplored area of research. The clinical trial was merely a proof-of-concept trial; the stability and delivery of the mRNA are still being tested (De Jong et al., 2019).

The Achilles heel of miRNA-based viral therapy is the lack of targeted miRNA delivery systems, off-target effects, and unidentified targets of miRNAs. In addition, miRNAs are relatively unstable, which may result in insufficient circulation and poor half-life of the miRNA-based therapy. Future research should be directed towards constructing optimal miRNA delivery systems and identifying methods to prevent off-target effects. As the use of miRNAs as treatment strategies is a growing field, only a few drugs have been FDA approved (Nature Biotechnology, 2020; Zhang et al., 2021b), which highlights the potential of RNAs for therapeutic intervention. MiRNAs provide a unique, reversible approach to treating human diseases and may be our secret weapon in our fight against HIV.

## 3 Conclusion

MiRNAs play a significant role in regulating gene expression. While the role of miRNAs in diseases such as cancer has been thoroughly investigated, the interplay between miRNAs and HIV infection has only begun to emerge. MiRNAs have emerged as key contributors to immune dysfunction observed in HIV disease. As research develops in specific subsets and more targeted populations, the understanding of this field matures as more can be uncovered. Considering that key genes involved in the HIV life cycle are affected by differentially expressed miRNAs, there is a link between the host’s RNA interference machinery and HIV pathogenicity. Future research should focus on identifying differentially expressed miRNAs in HIV-infected donors from different population groups., which may be exploited for therapeutic benefit.

In addition, the application of specific miRNA mimics and inhibitors (Andorfer et al., 2011; He et al., 2012; De Santa et al., 2013) is an appealing avenue for future investigations. Noting that one miRNA alone may be able to target several host genetic factors, the combined effect of several miRNAs together offers the potential for a multi-targeted effect. This treatment strategy can complement current cART regimen. Furthermore, inhibition of selected miRNAs is advantageous. For instance, selectively blocking miRNAs that target anti-viral proteins or pathways could potentially enhance anti-viral responses. This approach is efficient during the onset of infection, as the anti-viral response to HIV can be improved.
